# ‘We have the internet in our hands’: Bangladeshi college students’ use of ICTs for health information

**DOI:** 10.1186/s12992-018-0349-6

**Published:** 2018-03-20

**Authors:** Linda Waldman, Tanvir Ahmed, Nigel Scott, Shahinoor Akter, Hilary Standing, Sabrina Rasheed

**Affiliations:** 1 Institute of Development Studies (IDS), University of Sussex, Brighton, BN1 9RE UK; 2 International Centre for Diarrhoeal Disease Research, Dhaka, Bangladesh; 3 Gamos Ltd, Reading, UK; 4 The University of Newcastle, Newcastle, SA Australia

**Keywords:** College students, Gender, Mobile phones, mHealth, Sexual and reproductive health information, Gatekeepers, Disintermediation

## Abstract

**Background:**

Information and Communications Technologies (ICTs) which enable people to access, use and promote health information through digital technology, promise important health systems innovations which can challenge gatekeepers’ control of information, through processes of disintermediation. College students, in pursuit of sexual and reproductive health (SRH) information, are particularly affected by gatekeeping as strong social and cultural norms restrict their access to information and services. This paper examines mobile phone usage for obtaining health information in Mirzapur, Bangladesh. It contrasts college students’ usage with that of the general population, asks whether students are using digital technologies for health information in innovative ways, and examines how gender affects this.

**Methods:**

This study relies on two surveys: a 2013–2014 General Survey that randomly sampled 854 households drawn from the general population and a 2015 Student Survey that randomly sampled 436 students from two Mirzapur colleges. Select focus group discussions and in-depth interviews were undertaken with students. Icddr,b’s Ethical Review Board granted ethical clearance.

**Results:**

The data show that Mirzapur’s college students are economically relatively well positioned, more likely to own mobile and smart phones, and more aware of the internet than the general population. They are interested in health information and use phones and computers to access information. Moreover, they use digital technology to share previously-discreet information, adding value to that information and bypassing former gatekeepers. But access to health information is not entirely unfettered, affecting male and female students differently, and powerful gatekeepers, both old and new, can still control sources of information.

**Conclusion:**

Personal searches for SRH and the resultant online information shared through discrete, personal face-to-face discussions has some potential to challenge social norms. This is particularly so for women students, as sharing information may enable them to bypass gatekeepers and make decisions about reproduction. This suggests that digital health information seeking may be exercising a disruptive effect within the health sector. However, the extent of this disruption may depend, not on students’ mobile phone usage, but on the degree to which powerful new gatekeepers are able to retain control over and market SRH information through students’ peer-to-peer sharing.

## Background

Innovations in Information and Communications Technologies (ICTs), and especially mobile health, have been predicted to transform provider-patient relationships through the dissemination of health information, and by encouraging patient autonomy, self-management, and self-care [16]. People’s capacity to access health information is informed by power inequalities in health systems. Thus, while patients may know what symptoms they experience, they usually lack the medical knowledge for diagnosis and have to rely on health providers’ expert knowledge [12]. Physicians and other health providers also determine, and control access to, health care and treatment [16]. Health professionals thus serve a critical gatekeeping function. As health system intermediaries, they are particularly powerful, deciding on the flow of health resources (materials, medicines and information) and on what information is shared with patients. They thus ‘control the public’s knowledge of actual events’ ([34]: 144), acting as the ‘keeper[s] of medical knowledge’ and powerful decision-makers [16]. Bloom et al. [12] argue that health systems should be conceptualised as knowledge economies which exist to make clinical, medical, diagnostic and care expertise available to populations. This broader view of health includes all actors, both formal and informal, who provide expert health information. It thus draws attention to the wide range of health information and services which are also provided through markets, and incorporates analysis of economic and socio-political interests which underlie the different actors and institutions constituting the health knowledge economy. It furthermore emphasises health systems as concerned, not just with ill-health and disease, but with all activities that facilitate, restore and preserve health [13]. Such a perspective is appropriate for understanding college students’ health information seeking, which has blurred boundaries between health and medical concerns, and lifestyle and sexuality issues. A biomedical understanding of health and health information seeking does not necessarily highlight the emotional and other sexual health issues that college students are exploring (such as anxiety about or duration of sexual intimacy), nor does it address their lifestyle choices which impact on health (diet, fitness, skincare etc.). It also overlooks exposure to pornography in a range of sites (local magazines, imported videos). In this paper, health information refers to anything relating to the body and its wellbeing.

College students, as young adults in pursuit of sexual and reproductive health (SRH) information, encounter health system – and other – gatekeepers who control access to information [34, 54]). In Bangladesh, a culture of silence surrounds sexual relationships for unmarried young people, including college students, which means that many are hesitant to seek care even from health care providers. If they do decide to do this, the process is long and anxiety-filled [49]. This silence, and lack of health information, affects both young men and young women. Moreover, health professionals are not the only ones who perform a gatekeeping function. Teachers, parents, family members, older siblings and other community members may have access to information and resources which can be shared or withheld. They, like formal health gatekeepers, are influenced by a range of socio-economic and cultural factors, and use information and resources to promote or discourage particular behaviours [34]. This expertise, coupled with their status in society, enables them to exercise power and to provide or deny access to health services and resources based on a combination of personal knowledge, conservatism, resistance to changing cultural values, self-interest and finances.

The concept of a ‘digital divide’ captures the relationship between technological innovation, inequality and exclusion along the lines of gender, educational attainment and income [25]. Hilbert has, for example, argued that ‘Traditionally, longstanding inequalities prevent women from accessing ICT, leading to a vicious circle between digital exclusion, unemployment, low income and lacking education’ ([27]: 486). In keeping with this, male college students have been identified as those most likely to engage with digital technology [24, 35, 46],^1^ although as Hilbert [27] and Antonio and Tuffley [6] point out, when females do use internet technology, a range of benefits become possible. ICTs – particularly laptops and mobile phones with internet access – challenge existing forms of gatekeeping. They make health information (ranging from expert biomedical information, to experiential patient information and to unsubstantiated myths/claims) accessible and have the potential to undermine medical and socio-cultural gatekeeping practices that control college students’ access to information about sexual and reproductive activities. Access to ICTs thus enables college students to bypass medical and other gatekeepers of SRH information. College students’ readiness to engage with digital technologies (including Facebook) is readily apparent in Bangladesh with much focus on chatting, message sending, gaming, sharing videos, and posting [38, 47, 55]. Little attention has however been paid to the ways in which Facebook, or other service providers, facilitate discussions about health and health information dissemination and whether this differs for men and women college students.

Mobile phones and digital technology offer new ways of bypassing gatekeepers and innovative means of accessing health information, leading to what some commentators have heralded as ‘digitally engaged citizens’ – people accessing, using and promoting health information through digital technology unencumbered by intermediaries, and gatekeepers; a process termed disintermediation [17, 37]. This vision suggests that people such as these college students, should be able to use digital technology to access medical and health information, unfiltered by both formal and informal gatekeeping. It also suggests that, particularly in the area of sexual and reproductive health, this can have beneficial consequences for young people in terms of improving both male and female college students’ knowledge and understanding. In promising to bypass gatekeepers, mobile phones and other digital technologies may both echo and facilitate the liberating potential of medicine such as pills, injections, solutions. Geest and Whyte, building on Appadurai’s analysis of objects embodying particular values and having ‘social lives’, point out that ‘pharmaceuticals objectify the healing art of physicians and make it into some-thing that can be used by anyone’ ([19]: 348). These medicines – which are increasingly available over-the-counter in private pharmacies and unlicensed facilities – become things which embody the power and skill of medical specialists, through their highly-technological, curative, portable and saleable properties. These same properties also undermine medical professionals’ gatekeeping functions by offering people the promise, and perhaps the illusion, of being able to treat themselves.

This liberatory potential and the opening up of previously-controlled information, Eysenbach [17] hypothesises, will mean that traditional health system intermediaries and other gatekeepers are being superseded. Just as pharmaceuticals have enabled people to bypass health intermediaries, prioritising the ‘inherent power’ of drugs to heal ([19]: 34), phones and other technologies can be used to disseminate previously discreet information to young men and women. Health experts and other societal gatekeepers will no longer ‘stand between’ users and SRH information. However, users will still have to find ways of navigating the vast amount of information available to them through digital technology. Eysenbach proposes that gatekeepers will be replaced by other types of intermediaries who ‘stand by’, rather than between, users, helping them to identify relevant and accurate information. These new mediators, or apomediators, use digital technology to become educated about a particular subject, and then share this knowledge with peers as and when relevant [18, 37]. ICTs thus hold out a promise to eliminate traditional gatekeepers and, through a process of apomediation, to give users ‘direct, convenient access to an abundant amount of health information’, enabling them to share health information amongst themselves ([17]: 162). In so doing, this may have the potential to restructure health systems [16, 37].

Positive health system innovation extends beyond the public provision of health information through technology and the elimination of those gatekeepers whose access to knowledge and resources inhibits healthy and informed choices. The information provided has to be appropriate and any corresponding treatment efficacious. Health systems must retain the capacity to manage public health, while bearing in mind broader public goods and positive externalities that result from health system delivery. They need to accredit and regulate professional behaviour and ensure that medical providers, who may have their own prejudices and biases, do not exploit unequal power relations [12]. Ultimately health system innovation should ‘create the opportunities for health care organizations to build higher-quality, cost efficient and easily accessible health care organizations that are better adapted to the needs of their consumers with a lower overall cost’ ([11]: 49). This includes being aware of the ways in which digital technology opens up a world of readily-available health information, and of how processes of disintermediation and apomediation may change health information seeking in ways that impact on health service delivery.

### Digital health in Bangladesh

In Bangladesh, the health system comprises a complex mix of formal government and private facilities and a large and diverse range of informal providers, resulting in a range of experts and intermediaries [2, 26, 42]. The Bangladesh government has embraced the innovative potential of ICTs in the health sector, providing new ways of accessing health information and potential health benefits ([5]; Batchelor, et al., 2014). Mobile phones – and particularly bulk SMS messaging – have been used to disseminate health information since 2009 [20]; informing people about health promotion events such as national immunisation days [33]. In addition, the Directorate General of Health Services has redesigned its website to better communicate health information and makes use of Facebook, Twitter, and Google to attract citizens’ attention [20].

Mobile phones and internet use, particularly Facebook, have become increasingly widespread in Bangladesh [1, 55] allowing people to search for health information. Research has not, however, explored the ways in which young Bangladeshi college students use service providers, such as Facebook, for health information seeking or whether this differs for male and female students. There were 134 million mobile subscriptions in Bangladesh in 2015 [29]. This is roughly twice the number of unique subscribers as the GSMA reports 67 million unique subscribers in 2014 [23]. In 2014, Grameen phone reported five million active Facebook users [23]. That same year, Facebook launched Internet.org which allows people access to ‘25 government and private websites’ through mobile phones [7]. Phone credit is often sold as a ‘bundle’ which includes ‘free’ internet access, free phone calls, free calls to particular numbers, and cheap rates at certain times of day. Some bundles, designed to introduce and encourage social networking and online chatting, or to provide short-term access to Facebook, Whatsapp and Twitter, can be extremely cheap.^2^ Bundles for extensive phone, SMS and internet usage are more expensive.^3^

Many health initiatives rely on mobile phones [3, 4, 8] and commonly target community health workers,^4^ government health supervisors and managerial personnel, clinical health care providers, and women*.* Bangladesh’s six mobile phone companies operate health call centres which impart information about women’s health, smoking, alcohol or drug abuse, HIV/AIDS, immunization or nutrition. Health help lines provide medical advice, sometimes including prescriptions for over-the-counter drugs, and/or further referral [4, 9, 22].

Mobile phone access to the internet occurs alongside online advertising through social media for products such as vitamin tablets, saline solutions, pain-killers and products with less obvious health benefits (to increase hair growth, beauty products, skin lighteners or diet tablets). This is the context in which college students experience and encounter general health information. This paper contrasts their experience of mobile phones and health information with that of the general population in Mirzapur in order to explore innovation in health systems and asks whether college students in Mirzapur, Bangladesh are using digital technologies for health information in gendered and innovative ways.

Mirzapur Upazilla, located in Tangail district, is a predominantly Muslim, rural sub-district about 60 km northwest of Dhaka City. It covers an area of about 367km^2^ and had, in 2014, a population of just over 442,000 [40]. Mirzapur, which resembles other Bangladeshi sub-districts in terms of population and size, was chosen because of its semi-urban nature, with both rural and urban characteristics; the strong presence of all Bangladesh’s mobile network providers; the abundance of kiosks and other outlets providing mobile phone services and its proximity to Dhaka. Mirzapur’s residents are served by Kumudini Hospital, a not-for-profit private hospital, and a government Health Complex. Health information and services are communicated, and advertised, on television and radio, on posters and billboards, in pharmacies, in Kumudini hospital, or government offices and in village doctors’ premises,^5^ on mobile phones (through government-sent SMS), and through ‘miking’ (the public announcement of health services using megaphones).

## Methods

This study draws on data from two questionnaire surveys undertaken in Mirzapur by this research team. The first survey, undertaken between October 2013 and February 2014, drew a random sample of 854 households from the general population.^6^ The sample was designed to select about twice as many female as male respondents, with some 80% of respondents being either the household head or the spouse of the household head. Hereafter this is referred to as the General Survey. A second survey, referred to as the Student Survey, examined health information seeking and behavioural change among college students in Mirzapur. Undertaken in August 2015, it drew a random sample of 436 college students from two of Mirzapur’s largest colleges, namely Mirzapur Degree College and Government Saadat College, chosen because of high student numbers.^7^ The colleges are similar in terms of student numbers, both are government institutions which offer university-level education (from Higher Secondary School Certificate up to Masters-level degrees) and both are easily accessible by road. The survey was designed to select approximately equal numbers of men and women. Trained enumerators spent time at the colleges, recruiting survey respondents using a combination of non-probability sampling^8^ approaches based on convenience and snowballing. Students ranged from 17 to 28 years of age, with a median age of 21, and most students were unmarried (men 95%; women 76%). Survey respondents were grouped into socio-economic quintiles (poorest, poor, middle, rich and richest) based on the Asset Index developed by the Mirzapur Health and Demographic Surveillance System (HDSS) which in turn is correlated with the BDHS (Bangladesh Demographic and Health Survey) index.

The General Survey asked people about their ‘health’, ‘health information’ and ‘a serious health condition’. While it left open the definition of health, it offered a biomedical definition of a serious health condition (going to a hospital or using medicine for several months). This implied – and was interpreted to mean – a formal domain of health-seeking rather than more informal, personal notions of health. In the Student Survey, building on the results of the General Survey, we asked about both health and sexual health.

The surveys were complemented by four focus group discussions (two with women students and two with men), and in-depth interviews with college students (ten male and ten female), identified by the colleges as being good with smartphones. Men were interviewed by a male interviewer and women by a female. Students were asked to decide where the interview should take place. Many of the men opted to come to Kumudini Hospital (where we were staying), while most women students chose their homes. When interviewing women students, we were allowed to go into their bedrooms, but always the door was left open and senior women loitered outside. For these interviews, we identified students who owned smart phones as we felt they were the most likely users of mHealth. Interviews were translated and transcribed, before being coded according to key terms and analysed by themes.

Ethical clearance was obtained from the Ethical Review Board of Icddr,b. For in-depth interviews, informed written consent was obtained from the participants. For the focus group discussions and surveys, verbal consent was taken before commencing with the discussion. One limitation of this study lies in the design of the general population survey questionnaires, which did not ask about sexual health and which implied biomedical notions of health and ill-health, rather than personal notions of health.

## Results

This section compares mobile phone ownership amongst college students with mobile phone ownership amongst the general population of Mirzapur. It then explores the degree to which college students are interested in health information, whether they are more likely to use their phones to access health information and the extent to which such use is gendered. Finally, it examines college students’ and other Mirzapur residents’ use of technology for health information.

### Mobile phone ownership in Mirzapur

Table 1 shows the considerable difference between college students’ ownership and use of mobile phones compared with the general population. Whereas 55% of the Mirzapur general population owned mobile phones, over 90% of college students owned mobile phones and/or sim^9^ cards. Ownership among youth in the general population is not much higher than among the Mirzapur general population, suggesting that higher ownership among the student sample is linked to factors other than simply age. 56% of all students were intensive users, saying that they used a phone several times a day, compared with 39% of youth in the general population, also suggesting that intensity of use of mobiles is linked to more than age.

**Table 1:** Ownership of mobile phones and sim cards

	Student Sample	Mirzapur General Population	
	(*N* = 436)	Whole sample (*N* = 854)	Youth (18–24 years) (*N* = 92)
	Number (%)	Number (%)	Number (%)
Own a mobile phone	403 (92.4)	471 (55.2)	55 (59.8)
Own a sim card	406 (93.1)	473 (55.4)	57 (62.0)
Smartphone	208 (47.7)	n/a	n/a
Feature Phone*	184 (42.2)	n/a	n/a
Use mobile phone several times a day	243 (55.7)	244 (28.6)	36 (39.1)

*Feature phones are cheaper than smartphones and offer some, but not all, smartphone capabilities. These usually include touchscreens, text messaging, basic multimedia, internet capabilities, and access to social networking sites

All male students (99%) and most female students (86%) owned mobile phones. However, data from the qualitative research, revealed that while male students owned and carried their own phones around for personal use, female students owned phones, but their parents discouraged phone use, citing ‘their need to study'. Parental concerns also included time ‘wasted’ on phones, boys (or ‘unknown people’) phoning their daughters and relationships developing.

As Anita (who, like all respondents has been given a pseudonym) explains,

“*My parents do not want me to use the mobile phone right now, they do not like it…. if I started using mobile phone before... I would have spent time on it... I would have received phone calls, and attending to those calls would have been time consuming... Also I am girl... as a girl, you know, unknown people enjoy disturbing a girl by making phone calls during odd times! … This could hamper my study. So my mother did not allow me to have a phone until after my [Higher Secondary Certificate] examinations*”.

Similarly, Poly owned a phone, but her family did not allow her to use it until after her exams. Even when young women owned phones and were allowed to use them, they tended to leave them at home and, when asked, gave the socially-approved response that mobile phones were frivolous whereas they were hard-working students.

### Internet awareness in Mirzapur

Table 2 shows that 86% of the general Mirzapur population were unaware of the existence of the internet; yet among students only 5% were unaware. 64% of students claimed to have used the internet in the past month, as compared to only 3% of the general population. Although internet use was higher among youth from the general population than among the entire sample,^10^ it is still well below usage rates among the student sample, suggesting that age alone does not account for differences in internet use between the two samples. In the survey, most students (88.2%) indicated that they used their personal mobiles to access the internet.^11^

**Table 2:** Internet usage in Mirzapur in last month

	Student Sample	Mirzapur General Population	
	(N = 436)	Whole sample (N = 854)	Youth (18–24 years) (N = 92)
	Number (%)	Number (%)	Number (%)
Not aware of internet	23 (5.3)	737 (86.3)	51 (55.4)
Not used	133 (30.5)	92 (10.8)	34 (26.1)
Used internet in the last month	280 (64.2)	24 (2.8)	17 (18.5)

In qualitative interviews, college students explained that they had learnt how to use the internet or ‘*mobile browsing*’, as it was called, from friends, older brothers or cousins and, in the case of young women, their school teachers. Initially they played games, and downloaded music and videos. Their colleges also used the internet to communicate with them. Facebook (discussed in more detail below) is also a popular form of internet use. In Bangladesh, Facebook is necessary for college study activities as class schedules are posted here and it also offers newsfeeds, a source of jokes or other topics of interest, and ways to make new friends [38, 50].

Table 3, focusing on internet usage in the last month, shows that college students are much more likely to use the internet if they own phones. However, students without phones are more likely to be women. Internet users are more likely to own smartphones, although having a basic phone does not necessarily preclude access. Our survey results also show that smartphones are more commonly owned by male students (60% are owned by males, 40% by females, χ^2^(1) = 17.0, *p* = 0.000).

**Table 3:** College students’ internet usage in last month by gender, mobile phone ownership and type of mobile phone

Variables	Internet user (*N* = 280)	Internet non-user (*N* = 156)	*p*-value (Pearson Chi-square)
	Number (%)	Number (%)	
Gender			
Male	185 (66.1)	32 (20.5)	0.000
Female	95 (33.9)	124 (79.5)	
Phone ownership	274 (97.9)	129 (82.7)	0.000
Types of Phone			
Smart phone	175 (62.5)	33 (21.2)	0.000
Feature phone	110 (39.3)	74 (47.4)	0.099
Basic phone*	10 (3.6)	24 (15.4)	0.000

*It is not clear how this small number of respondents accessed the internet

Internet usage among college students is gendered. When asked about usage in the past month, men were twice as likely as women to have done so (Table 3). This is echoed in the qualitative findings showing general societal disapproval of young women students using their phones and/or the internet, as this would encourage inappropriate behaviour, or as one respondent explained, ‘*he [my father] said if I use Facebook, I would walk in the wrong path*’. However, as discussed further below, both male and female students report using mobile phones in ways which demonstrate potential for innovation and disintermediation. This suggests that women college students are able, at least to some extent, to bypass societal controls on their mobile phone usage.

When asked how they had used the internet in the past month (Table 4), students cited Facebook as their primary activity (95%), followed by googling (46%), online chats (30%) and downloading/listening to music (29%). In Bangladesh, Facebook data is included as part of a ‘phone credit bundle’ and students thus talk of ‘free internet’ or ‘free Facebook’. Table 4 shows that overall male college students tend to use more internet services. Almost all college students used Facebook, available in Bengali, ‘*to chat to friends*’, and as one informant told us, to be “*more connected through these. It’s comfortable and a lot of people appreciate it*”.

**Table 4:** Use of Communication Services in past month by College Students

Internet activity	Male Number (%), (*N* = 185)	Female Number (%), (*N* = 95)
Googling	95 (51.4)	35 (36.8)
Email	25 (13.5)	3 (3.2)
Music	55 (29.7)	27 (28.4)
Videos	37 (20.0)	10 (10.5)
Chat	49 (26.5)	35 (36.8)
Facebook	175 (94.6)	90 (94.7)
Skype	13 (7.0)	8 (8.4)
Twitter	8 (4.3)	0
News/Sport	61 (33.0)	11 (11.6)
Keep in Touch with Family	24 (13.0)	9 (9.5)
Other	4 (2.2)	0

*This included health forums

### Using technology to acquire health information in Mirzapur

Although students are using mobile phones and the internet far more than the general population of Mirzapur, only 12% of students surveyed reported having used the internet specifically to search for health information. Most common usage of the internet was through Facebook and Google (Table 5). Although sub-sample numbers are small, Google appears to be the preferred internet service for searching for health information by women college students. This may reflect their desire for anonymity or it may be that they are searching for health information not available through other services like Facebook. Only one person recalled using a health forum in the past month. However, this apparently limited use of the internet for health information seeking partly reflects the way the survey was constructed and does not mean that these students were uninterested in health-related information. As one college student explained: “*on my Facebook, with my ID there are a lot of health pages. I like them. Whichever comes in front of me, whenever I find this is a health page or treatment page I will ‘like it*”. Other students ‘liked’ and read pages on heart attacks, home treatments for coughs, or flu symptoms. One student ‘liked’ the posts his friends ‘liked’. Through Facebook, accessed ‘free’ as part of their credit bundles, they could visit these health-related websites: MAMA,^12^ Maya.com.bd,^13^ HealthPrior21.com^14^ and UNICEF.bd.^15^ Some students – but only male ones – also noted opportunities to watch erotic material or pornography.

**Table 5:** Most commonly-used Internet services when searching for information on health

Students who have used the internet to search for information on health	Male Number (%), (*N* = 38)	Female Number (%), (*N* = 16)
Facebook	26 (68.4)	7 (43.8)
Searching (googling)	18 (47.4)	11 (68.8)
Health forums	7 (18.4)	1 (6.3)

Table 6 shows that, in the past month, college students had used a range of media formats which provided them with health information. Of these, newspapers had the greatest reach, with 31% of all respondents recalling reading health information. Comparing youth from the student and general population samples shows that students are more likely to see information in printed forms (newspapers and printed materials); it is not clear whether this is simply due to literacy, or whether printed materials are more commonly found in the college environment. College students also recalled getting health information (diet and nutrition; beauty; HIV/AIDs; diarrhoea; non-specific childhood illness; fever) in the form of SMS messages, Facebook or Google from mobile phones (29%), in the form of health entertainment, public health messaging and, most frequently, commercial adverts on television (26%) and in the form of printed messages in newspapers, advertising pamphlets etc. (25%). This contrasts with the general Mirzapur population where television (30%) and public miking (35%) were the primary sources of health information.

**Table 6:** Students’ health information sources in the last month compared to general population

Medium	Student Sample,	Mirzapur General Population	
Heard health message (% of all respondents)	(N = 436)	Whole sample (N = 854)	Youth (18–24 years) (N = 92)
	Number (%)	Number (%)	Number (%)
Radio	20 (5)	15 (2)	5 (5)
Television	115 (26)	258 (30)	37 (40)
Newspapers	134 (31)	24 (3)	6 (7)
Printed messages	110 (25)	89 (10)	15 (16)
Music/dance/drama	69 (16)	26 (3)	6 (7)
Miking	98 (23)	296 (35)	34 (37)
Mobile phone	126 (29)*	33 (4)	12 (13)

*Received unsolicited SMS regarding health

In the qualitative interviews, students identified a wide variety of health information sources including radio, television, newspapers, Kumudini hospital, health clinics and health help lines. They also all knew of the internet as a potential source of health information, and several had browsed in search of specific information, such as the availability of specific treatments from hospitals. In addition, they received health messaging during daily phone usage such as health tips and prevention, fitness regimes, dealing with common ailments and disease-specific information (dengue or swine flu alerts). Women college students also mentioned searching for SRH information on menstruation, pregnancy, and contraception; while men students emphasised topics such as HIV, and sexual intercourse. Internet use avoided their embarrassment of ‘*problems that we can’t tell in front of others*’, not least because: ‘*if I ask [health providers or other gatekeepers] something who knows what they’ll think, but the internet won’t think anything. The internet is for giving information*’. Using phones and the internet in this manner echoes the privacy provided when medicinal drugs are treated as ‘things’ liberated from medical regulation and specialist oversight and can, as such, be obtained as ‘common commodities … purchased like other daily wares in shops and markets’ ([51]: 123). Medicine in this form allows people to take control of their conditions, particularly if these conditions have negative connotations (or, in the case of young people, contravene societal norms) and to maintain privacy [19]. Phones, like pharmaceuticals, have the potential to bypass medical authority and, in so doing, restructure the relationship from patient to consumer.

### Use of mobile phones and internet for health information

Table 7 shows that, in the past year, students have been more likely to use mobiles for health information than the general population. Slightly less than half the students (45%) recalled that they had used their phones for at least one health-related purpose in the past 12 months. This contrasts with 18% in the general population. In comparison with youth from the general sample, it is evident that college students’ increased use of phones for health purposes is due to more than age. Youth in the general Mirzapur population are less likely to call a village doctor or doctor, and they are more likely than college students to use the phone to get advice (from an unspecified person, such as a family member, friend, or other personal contact).

**Table 7:** Use of phone for health purposes (in the last year)

Used Mobile Phone for Health	Student Sample	Mirzapur General Population	
	(N = 436)	Whole sample (N = 854)	Youth (18–24 years) (N = 92)
	Number (%)	Number (%)	Number (%)
Contacted Village Doctor by Phone	80 (18.3)	48 (5.6)	2 (2.2)
Contacted Doctor	76 (17.4)	73 (8.5)	13 (14.1)
Contacted Health Worker	31 (7.1)	12 (1.4)	3 (3.3)
Contacted Other Health Professional*	15 (3.4)	31 (3.6)	3 (3.3)
Got a Serial Number**	80 (18.3)	34 (4.0)	6 (6.5)
Tracked a Doctor***	43 (9.9)	28 (3.4)	5 (5.4)
GotAdvice on Condition/Treatment	37 (8.5)	66 (7.7)	14 (15.2)
Made a Complaint to a Healthcare Provider	11 (2.5)	6 (0.7)	0 (0.0)

*This refers to pharmacists, private doctors, etc.

**A local term for a doctor’s appointment

***This refers to checking whether a doctor is at his practice or not

In qualitative interviews, students identified a wide range of health information needs, including: how to maintain healthy lifestyles, good skin and hair conditions; how to deal with other family members’ or neighbours’ health issues (such as kidney problems, or needing specialist health services); how to manage menstruation; sexual relations after marriage and how pregnancy happens. College students most common health-related phone activities were to contact informal healers such as village doctors (18%), contact medically-trained doctors (17%) and make appointments with doctors (also 18%). Amongst the general Mirzapur population, the most common use of mobile phones for health purposes was to contact a doctor (9%) and to get advice on a treatment or condition (8%), although considerably fewer did this than in the student population. College students also used their phones to get advice on a condition or to secure treatment (9% as compared to 8% in the Mirzapur general population survey). This suggests that students are interested in using mobiles to access health help lines and new ICT-based government health services.

Nonetheless, internet usage remains low amongst college students because of the lack of bandwidth, lack of internet-connected computers and laptops, poor network coverage, lack of 3G, and frequent network collapse, all of which undermine the value of online searches. Indeed, as one student commented ‘*by the time we search for something and the results come up, we could have reached the hospital*’, suggesting it was faster to get professional help than to go looking online for health information.

There is evidence that college students use the internet when they need broader health information. A higher proportion of students who had cause for concern (worried that they might have had any kind of health problem)^16^ had used certain media to search for health information in the past year (Table 8). 17% had searched the internet, compared with 8% of students who had not had any worries about their health. Students who had cause for concern were not more likely to watch television, but they were more likely to recall having seen health-related spots or programmes on television. This trend was not evident for health messages in radio or newspapers, but students with health concerns were also more likely to have spotted some form of printed materials on health (undefined, but including posters, flyers, adverts etc.).

**Table 8:** College students’ search for health information (comparing those with, and without, cause for concern in the last year)

Variables	Worried (in the last year)		Not worried (in the last year)		*p*-value (Pearson Chi-square)
	N	Number (%)	N	Number (%)	
Used internet service for seeking health info (at least 1)	187	31 (16.6)	197	15 (7.6)	0.007
Heard radio spots or messages on health (in last 30 days)	65	6 (9.2)	76	13 (17.1)	0.172
Seen TV spots of programmes on health (in last 30 days)	181	60 (33.1)	189	41 (21.7)	0.013
Read health messages in newspapers (last 30 days)	187	68 (36.4)	197	59 (29.9)	0.182
Seen other printed health messages (in last 30 days)	187	61 (32.6)	197	34 (17.3)	0.000
Heard miking messages on health (in last 30 days)	187	47 (25.1)	197	35 (17.8)	0.078

In interviews and FGDs, all college students, both male and female, spoke about their interest in skin care, fitness and diet and how the internet gave them access to this health information. Not all the information received was biomedical. For example, worried about the condition of his hair (slow growth and thinning), Ishrat used Google and followed advice suggesting he apply a mixture of castor and coconut oil. This, he believed, solved his problem. Johnny ‘*found something on Facebook*’ saying that garlic is beneficial for asthma and told his friends and family. ‘*Now everyone’s having garlic and getting benefits*’ despite not suffering from asthma. In these instances, there is scope to use non-biomedical health information, but this may be less appropriate if faced with more serious health conditions (discussed in more detail below). Both men and women students also browsed for information on sex, and what to eat to keep fit. Women students were interested in understanding reproduction and controlling fertility, while male students looked at pornography and searched for information on prolonging intercourse. As one male informant explained, ‘*well I have searched a bit [for SRH information]. Now if you talk about pornography, even people 5 years younger than us watch porn regularly… They’re watching it on the phone, on the laptop, it’s everywhere*’.

Some male students had used the internet for other family members’ serious health problems. Ishrak made an online medical appointment at a hospital in Dhaka for his mother’s eye problem. Johnny identified ‘*good*’ and ‘*well-reputed*’ doctors online after his mother’s car accident, and for his grandfather’s kidney problem (however, financial limitations prevented any uptake). Shafwat tried (unsuccessfully) to call a health help line to enquire about his younger cousin’s loss of appetite.^17^ Saad googled for information after his younger brother was diagnosed with a tumour and collected ‘*everything*’. With the exception of Ishrak, none of these students acted on the online information and seldom shared it with their families. As a consequence, online health information did not usually translate into particular health seeking behaviours.

Female students (most of whom were unmarried) searched online to find out about their bodies and sexual health. They spoke of their difficulty discussing these issues with doctors (particularly but not exclusively male) and other adult women. Even though societal norms discouraged young women students from accessing SRH information and from ‘frivolous’ mobile phone usage, these students emphasised their newfound ability to use their phones (often without their parents’ knowledge or permission) to avoid gatekeepers. Some searched for information on menstruation. Fatima for example, had ‘*no idea about it*’ and said, ‘*I knew about it when I first experienced it… I was not informed by anyone [about menstruation]*’. Others, like Bithi, researched menstrual pains for herself and pregnancy advice for another. Nisha Googled contraception.

## Discussion

The data show that Mirzapur’s college students are economically relatively well positioned, more likely to own mobile phones, and more aware of the internet than the general population and youth in the general population (Tables 1–4). These college students are interested in health information and are more likely to use their mobiles and, where available, computers to access this information (Tables 5–8). Does this mean that ICTs are the tools through which health systems are undergoing innovation and change? Are ICTs facilitating an emergent process of disintermediation and apomediation in Bangladesh’s health system?

The concept of disintermediation imagines a process of unmediated access to information, which enables people to navigate around gatekeepers, retrieving information which is usually controlled by professional interest groups of various kinds. Evidence from Bangladesh, however, points to the importance of social and cultural gatekeeping in a conservative society, with constraints (stricter for women and unmarried men) on young people’s sexual health information. Young people trying to get information on issues such as sexual and reproductive health thus often have to overcome barriers imposed by a wide range of health gatekeepers, including family, elders, guardians of socio-cultural knowledge, health system providers and medical experts, as the keepers of medical knowledge and what it is ‘appropriate’ for them to know, and as decision makers about access to services and treatment. The reality in Mirzapur is therefore far from a utopian world of disintermediation and apomediation. While both male and female students looked for health information online – from how to care for one’s skin to what to do about tumours – and were able to access some information, there were limits to what information they accessed and how they could use it. Lupton, focusing on ‘digitally-engaged patients’, argues that digital technology and disintermediation should provide the means of ‘preserving and promoting their own good health, including accessing relevant information, monitoring their own health and taking responsibility for managing their medical conditions’ ([37]: 857). In the case of college students’ online health information seeking, it is however clear that practical, technological, gender and cultural barriers created considerable limitations.

Nevertheless, in Mirzapur, access to digital technology offered college students ways to evade some gatekeepers’ social control and, in so doing, to make health and SRH information more freely available to them. Sexual health is an area where gatekeeping is particularly prevalent. Conventionally information about SRH was seldom shared between adults and students, and particularly not between adults and young women ([48]: 169; [28, 31, 49, 52]). In our research, male and female students were accessing and sharing SRH information. This enabled some women students to gain a better understanding of their bodies and a sense that they had more control over their fertility. For example, Nisha, a young married woman, wanted to know what form of contraception was most appropriate. As she was too shy and uncomfortable to approach a doctor and was confused about the information she had received through conventional health information sources (miking, posters, television, newspapers), she turned to the internet, learning how a woman’s body operates and how an embryo evolves. She read that, if she avoided intercourse 7–12 days after menstruation, then she did not need to use any contraception. For Nisha, this was better than other sources because it provided her, in her estimation, with a ‘*full or entire analysis*’. While access to this information provided Nisha with some reassurance, it may not have been a complete and medically-accurate analysis and there are few guarantees that she will be able to delay pregnancy as a result of this knowledge.

There are, of course, potential disadvantages associated with the bypassing health professionals and other traditional gatekeepers. Van der Geest and Reynolds Whyte [19] examine the consequences of this in relation to drugs and medication. They point out that people who adopt ‘self-treatment’ are dependent on impersonal market relations and vulnerable to exploitation by other, less medically-qualified persons – such as informal drug vendors, shop keepers, etc. – who, while advising on pharmaceutical consumption, may not have patients’ best interests at heart. Similar issues are at stake in relation to mobile phones and access to health information, reflecting both the need for a broader health knowledge economy approach [12] and the need to consider how currently unregulated ICT-mediated health information can make ‘unsubstantiated claims to medical authority’, or ‘misleading claims’ or suggest ‘inappropriate treatment’ that may ultimately cause harm ([13]: 6,7). These and other risks are evident in the college students’ navigation of health information, including that the students will not be able to identify effective, accurate suppliers of information; that the information and health solutions found online will not produce the results desired; that they will spend money on ineffective treatments and that they will misuse drugs resulting in increased threats to their health and public health more broadly. At present however, the risks experienced by Mirzapur’s college students are relatively low. This is because, first, the kinds of health information – and consequent treatments – being pursued by Mirzapur’s college students tend to focus primarily on skin, fitness and beauty rather than severe illness or disease. Secondly, these students are not at present purchasing drugs online and thus their actions do not have high risk potential. Recall, for example, Johnny’s recommendation of garlic for asthma. This is unlikely to have major negative consequences and, if instances of asthma continue, affected persons are likely to seek further information and treatment. Thirdly, despite challenging societal norms and bypassing conventional gatekeepers, through unmediated internet access, college students’ use of inaccurate health information may have contradictory consequences. Take for example Nisha’s search for appropriate information about contraception. For young married women such as Nisha, the expectation is that they should bear children soon after marriage, so the consequence of poor SRH information on avoiding pregnancy is not likely to be of concern, except for the young women themselves. The consequences of inaccurate contraceptive information, if coupled with sexual activity, would however be far more significant for unmarried women [28]. Moreover, the long-term consequences of using mobile phones to access a wide range of health information should not be under-estimated. Hampshire et al. [24] have argued that, as new forms of technology create new health markets, so young people and others will be increasingly targeted and they will have to develop appropriate resources, networks and skills to navigate their way through these markets.

To date, however, access to the internet, usually through mobiles and occasionally computers, has provided male and female students in Mirzapur with discretion and anonymity, factors which, for youth, may be more important to them than health providers’ qualifications [32] and biomedical accuracy. In private, female students searched for information about their menstrual cycle and pregnancy, while male students searched for topics like HIV, the use of condoms and sexual performance. Their more general health interests – how to stay healthy, avoid disease and so forth – were publicly shared, as the students ‘liked’ health pages and Facebook feeds. Even this seemingly minor act of ‘liking’ a page, and promoting it amongst peers has, as Lünich and colleagues argue, a gatekeeping function in that it rates and promotes particular websites’ and certain kinds of information [36].

Such actions facilitate apomediation, the process by which college students use digital technology and, having become educated about their bodies and health conditions, share knowledge with their peers, and provide information to family members when relevant [37]. Apomediation is, in theory, different to gatekeeping because it does not reinforce hierarchical power relations. While gatekeepers are seen to stand ‘in-between’ people and health information and treatment, apomediators are understood to ‘stand by’ their peers and others in need of health information ([17]: 162). Apomediators have the choice whether to share information, when to share it and in what format, but with few guarantees about the accuracy or efficacy of this information. Bithi, helped her newly-married sister who had experienced two successive miscarriages, by downloading a health app containing pregnancy information and instructions on activities to avoid. Other girls shared information on menstrual hygiene and not getting pregnant with close friends. This kind of information sharing is highly significant in a society where college students, and indeed all young people’s but especially young women’s, sexuality has been carefully monitored, and SRH information strictly controlled by gatekeepers, because it gives these students access to information which helps them understand more about their bodies and their fertility and thus to make more (but not necessarily wholly) informed decisions about intercourse, sexual health and reproduction.

In theory disintermediation and apomediation provide college students with greater choice as to who might offer health information. Some may continue to use the intermediaries they have always used. Others might look to new intermediaries. The tendency to look for new intermediaries, Eysenbach suggests, is particularly pronounced among young people who, ‘strive to become more autonomous and to reduce the power of intermediaries such as their parents, with peers taking on the role of the former intermediaries’ ([17]: 162). Older adolescents seek to challenge authority, strive for greater autonomy and find peer-to-peer apomediation attractive.

Our study shows that willingness to engage in disintermediation and apomediation is influenced by age, gender, health condition and by familiarity with digital technology. Despite young men having greater access to digital technology, college students of both genders are using phones and computers to explore sexuality and healthy lifestyle choices. This information is relatively easy to act upon, not requiring significant social and financial resources. Mirzapur’s students found online information to be useful and engaged, to some extent, in peer apomediation, sharing health information in face-to-face discussions with friends and similarly-aged relatives who were not as experienced online, and through ‘likes’ and forwarding posts.

Familiarity with technology also influences the extent to which college students use digital technology as a means to bypass intermediaries. Thus, the more intensively students used mobiles, the less likely they were to defer to their parents as the primary health intermediary. There is, as shown above, a dominance of male students among internet users, reflecting both lower levels of familial gatekeeping and their greater access to the internet. This, Eysenbach [17] suggests, creates potential for a feedback loop; as students get better at accessing information, there is more reliance on peers to share information.

There are also new intermediaries, such as digital providers and college teachers that – whilst embedded in conventional socio-cultural systems and power relations – allow the possibility of bypassing gatekeepers. College teachers are required to teach SRH in schools [52]. Socio-cultural norms, shame and fear of stigma which prohibit the sharing of this information across age and gender categories make this an embarrassing experience for teachers and students alike. As a result, some teachers have avoided teaching this and have instead referred students to the internet. This has particularly influenced women college students. Shahinoor’s teacher, for example, explained: ‘*If you have any problem, you will find a separate page after logging into internet*’. So, when Shaninoor was interested to find out more about contraception, she remembered ‘*Sir told us that if there are any health or questions like that… Sir gave us an address, so I searched by typing that*’. At the time of the interview, Shahinoor had never searched on Google, she had only used the website address given to her by her teacher. Bithi’s teacher, equally embarrassed to teach biology, had said ‘*you can find anything if you Google it*’ and thus encouraged her to explore health and reproductive information online. Bithi then searched for health information online, finding information and downloading health apps.

Digital service providers in the telecommunications industry also act as new gatekeepers to health information through the types of internet access and bundles they offer. Social network providers such as Facebook provide information to members through, for instance, selected links which direct college students, and other young people, to particular websites. In so doing, Facebook offers an illusion of using the whole internet, but this is limited to particular sites. As indicated in the following quotations, at least some of Mirzapur’s college students, both male and female, do not recognise this distinction and associate internet searching with Facebook:

Interviewer: ‘*How long have you been using the internet’?* Respondent: ‘*I’ve basically been connected to the internet because of Facebook. Since 2012, it could be said’*.

Interviewer: ‘*So which medium do you use to get information’?* Respondent: ‘*… for the exchange of information I use the internet, mainly Facebook browsing’*.

This limited access undermines the concept of the internet, which is to enable anyone to connect up to anyone else without gatekeepers and without permission. ‘The internet by definition is a vast collection of interlinked sites – over a billion of them, at last count. And Facebook offers about 0.0000002% of this to the user’ ([41]: no page number; [53]). The implications are significant, as Facebook is Bangladesh’s most popular internet service, used by 64% of students in Mirzapur.

Given the limited internet literacy and the heavy reliance on mobile phones to access the internet, there is little scope or incentive for college students to go beyond these sites. This gatekeeping function is of particular consequence not only because it is not immediately apparent, but also because, as Facebook is a commercial platform, its users’ interests are exploited to sell targeted advertising space. This can turn a user’s passing concern with, say baldness, into a targeted marketing strategy directing the user to a range of commercial, rather than efficacious or appropriate, medication and, as suggested above, making users vulnerable to unscrupulous market exploitation that does not have their health interests at heart. In addition, while Facebook does provide access to four health-related websites, college students are more inclined to ‘like’ informal, unregulated health information – such as Johnny’s reporting of the benefits of garlic described above. This informal, unregulated health information is, as Eysenbach suggests, an important aspect of apomediation, with experience-based information and personal accounts of efficacy assuming increased significance and credibility. The incorporation of digital technologies in health systems may thus mean that, while ‘former intermediaries do not disappear completely’ ([17]: 165), new gatekeepers give the appearance of being apomediaries, obscuring commercial interest behind a façade of peer-to-peer mediation.

## Conclusion

Almost two decades ago, Goldsmith predicted that ‘the internet has a greater potential to fundamentally transform both the structure and the core processes of medicine than any new technology we have seen in the past fifty years […] The most important effect of the internet will be to strengthen the consumer’s role in relation to practitioners and health care institutions, and to create a powerful new tool to help people manage their own health risks more effectively’ ([21]: 155–6). This paper shows that mobile phones and computers have created new opportunities for college students in Mirzapur to access and to share health information which was previously controlled by health intermediaries and other gatekeepers, and often discouraged by socio-cultural values and norms. Despite predictions that ICT innovations will transform patient-provider relationships and health systems, access to health information in Mirzapur was not unfettered. Social and cultural control through senior family members and other cultural gatekeepers of access to digital sources of information continues to be very strong, particularly for young women. At the same time, new intermediaries such as school teachers and some commercial digital platforms have played a more enabling role in encouraging access. But this has been at the price of determining what sources of information to reveal or to restrict, as the case of service providers like Facebook show.

Disintermediation has taken place to varying degrees with both male and female students searching online for health information. The ways in which college students share information online, through ‘likes’, forwarding posts etc., is more in keeping with what one might expect in this era of digital innovation. Yet it is perhaps the personal searches for sexual health, undertaken by both women and men, and the information found online but shared through discreet, private and personal face-to-face discussions, that most challenges social norms and is perhaps most innovative. This is perhaps most radical in the case of some young women students because it provides them with information which facilitates decisions about reproduction that were previously in the hands of senior women, men and health service gatekeepers.

Using mobile phones to access the internet was something college students were doing in relation to sexual health and lifestyle information, and was an activity which enabled them to bypass intermediaries and gatekeepers; but was not an activity undertaken in critical health situations. To what extent then, does this digital health information seeking portend future innovation and disruption in the health system?

The evidence of disintermediation and apomediation suggests that changes in access to health system knowledge and power relations are likely but will still have to contend with the power of social and cultural norms. There is evidence that familiarity with digital technologies enhances the likelihood that conventional gatekeepers will be circumvented and that, even as new intermediaries emerge, new sources of information will be promoted which are less grounded in socio-cultural norms and power relations, but access is still likely to be gendered and to be subject to policing by family members or by other social and political forces. The data suggest that apomediation and experience-based credibility may become increasingly desirable and relevant but that this will raise important questions about the quality and reliability of information and how these are ‘brokered’. Because these college students are likely to continue using digital technology as they age, improvements in their technological skills, in the technology itself, and in access to the technology are to be expected. Ultimately however, large-scale health system innovation may depend not on the innovations used by college students, but rather on the degree to which powerful internet gatekeepers – such as Facebook – are able to dominate control over SRH information while marketing seemingly unmediated health information and treatments in the guise of peer-to-peer apomediation.

## Abbreviations

ICTs: Information and Communications Technologies
SRH: Sexual and Reproductive Health
HIV/AIDS: Human Immunodeficiency Virus Infection and Acquired Immune Deficiency Syndrome
SMS: Short Message Service

## Footnotes

1 Young, educated and better-off males tend to be most active on the internet or most experienced with mobile phone usage [10, 30] with evidence from both high income countries [10, 15, 35, 39, 44] and low- and middle-income countries [14, 45]. For example, Nisbett shows that young men in Asia enthusiastically embraced careers in IT enterprises and adopted ‘highly visible consumption habits’, exemplified in mobile phone use ([43]: 175).

2 In February/March 2016, Grameen phone offered 20 min talk time, 5 MB internet usage, 5 SMS and 5MMS over a two-day time period to Grameen subscribers for 5 Taka. Banglalink advertised ‘Facebook, Whatsapp & Twitter All-Day Long’ for one Taka.

3 Airtel offered users 2400 min, 10,000 SMS messages and 10GB internet access for 2000 BDT per month in March 2016.

4 Community health workers or *Shasthya Sebikas* are trained workers who support Upazila health complexes by undertaking home visits to promote health and provide family planning services. Health workers can be Non-Government Organisations’ employees, voluntary community workers or government employees.

5 These informal healers were introduced in the 1970s and modelled on the Chinese concept of barefoot doctors. These village doctors have received limited training, in the form of short courses run either by the government or pharmaceutical companies.

6 In the absence of prior variance estimates of the outcome variables, a value of 0.5 (the maximum for dichotomous variables) was used to calculate the required sample size to obtain 95% confidence limits with a precision of ±10%, assuming a design effect of 2. This implied a sample size of 840 households.

7 The target sample size for the college students was 420 because in the absence of clustering, a design effect of 1 was assumed.

8 After formal introductions with the college head, researchers were directed to possible participants and thereafter used personal introductions to peers and opportunistic meetings. There is no reason to believe that selected students were atypical in respect of their ICT use and sample size was large enough to minimise bias.

9 A small plastic card with an embedded microchip storing information: user identification, phone number, credit, network authorisation, and contact numbers.

10 For the purposes of comparison data from the General Mirzapur Population survey has been presented along with a sub-sample representing youth. The age bracket of 18–24 years has been selected on the basis that 97% of the college student sample fall within this range.

11 Other sources included internet cafés (2% (*n* = 6); fixed modems (9% (*n* = 26); places of work (3% (*n* = 9) and other (% (*n* = 10).

12 Launched in 2011, MAMA (Mobile alliance for Maternal Action) or Aponjon as it is known locally in Bangladesh is a public-private partnership involving the Bangladeshi Ministry of Health and Family Welfare and the Prime Minister’s Office, Johnson and Johnson, USAID, the UN Foundation, and BabyCenter. It is delivered by D-Net, DNet, a social enterprise that employs ICTs to encourage knowledge and access to information. MAMA it uses mobile phone technology and SMS messaging to provide free, stage-appropriate, expert health information to pregnant women and new mothers. http://www.aponjon.com.bd/mama_inner and http://www.unfoundation.org/what-we-do/issues/global-health/mobile-health-for-development/mama.html, accessed 2 December 2017.

13 Founded in 2011, this Bangladeshi technology company connects women and young people to a wide range of health information and provides answers for health questions seldom publically voiced through its website, its app and internet.org. http://www.maya.com.bd/#/about accessed 9 March 2016

14 Billed as ‘Asia’s first total health care solution Portal and Health Care Management Centre’, this health portal allows patients to read health advice, request consultations with doctors and purchase products. http://www.healthprior21.com/about-us accessed 9 March 2016

15 The United Nations Children’s Fund (UNICEF) was established 70 years ago and now operates in 190 different countries and territories. It protects children’s rights, helps to ensure basic needs and seeks to expand children’s opportunities to achieve their full potential. The UNICEF Facebook page operates as an ‘engaging and learning environment for our fans to share ideas and to discuss UNICEF, UNICEF’s work and the rights of children worldwide’. https://www.facebook.com/pg/unicef.bd/about/?ref=page_internal accessed 2 December 2017.

16 This is does not include students who may have had ‘serious’ health concerns.

17 The call was answered and he was presented with different options, to narrow down the type of problem that he was calling about. After selecting the relevant option, ‘at one point it got disconnected. So I didn’t call again’
